# Increasing Incidence of Nontuberculous Mycobacteria, Taiwan, 2000–2008

**DOI:** 10.3201/eid1606.100228

**Published:** 2010-06

**Authors:** Eduardo Hernández-Garduño, R. Kevin Elwood

**Affiliations:** British Columbia Centre for Disease Control, Vancouver, British Columbia, Canada

**Keywords:** Nontuberculous mycobacteria, bacteria, disease, colonization, Taiwan, letter

**To the Editor:** Lai et al. (*1*) reported an increase in the number of nontuberculous mycobacteria (NTM) isolates and patients with pulmonary NTM diseases after implementation of the BACTEC system (Becton Dickinson, Sparks, MD, USA) late in 2001. These authors also reported that the increase was mainly in persons infected with *Mycobacterium avium* complex (MAC) and *M*. *abscessus*. They stated that diseases caused by NTM were defined according to current diagnosis criteria published in 2007 (*2*). This finding suggests that Lai et al. were able to review the clinical and radiologic information for all patients.

We wonder whether they were also able to identify and exclude people with NTM colonization, i.e., persons with positive cultures for NTM who did not meet the American Thoracic Society disease criteria. It would have been interesting to know the trend in colonized persons. In a previous study from British Columbia (*3*), we found an increase in the number of NTM isolates mostly in persons with MAC colonization. This finding coincided with implementation of a new laboratory technique in 2000, which suggested that the new technology is more sensitive in detecting MAC. In contrast with the findings of Lai et al., our study from British Columbia showed that the incidence in patients treated for NTM pulmonary disease (the group used as a surrogate of NTM disease) has been decreasing over time, which is reassuring.
