# Supplementary material for: Increasing Incidence of Nontuberculous Mycobacteria, Taiwan, 2000–2008
Source: Emerg Infect Dis. 2010 Jun;16(6):1047–8. doi: 10.3201/eid1606.100228 (PMC3086241; doi:10.3201/eid1606.100228)
Supplement: Related texts

# Letter: Increasing Incidence of Nontuberculous Mycobacteria, Taiwan, 2000–2008

**DOI:** 10.3201/eid1606.100329

**Published:** 2010-06

**Authors:** Chih-Cheng Lai, Che-Kim Tan, Chien-Hong Chou, Hsaio-Leng Hsu, Chun-Hsing Liao, Yu-Tsung Huang, Pan-Chyr Yang, Kwen-Tay Luh, Po-Ren Hsueh

**Affiliations:** Cardinal-Tien Hospital, Taipei, Taiwan (C.-C. Lai); Chi-Mei Medical Center, Tainan, Taiwan (C.-K. Tan); National Taiwan University College of Medicine, Taipei (C.-H. Chou, H.-L. Hsu, Y.-T. Huang, P.-C. Yang, K.-T. Luh, P.-R. Hsueh); Far Eastern Memorial Hospital, Taipei (C.-H. Liao)

**In Response:** We thank Hernández-Garduño and Elwood for drawing attention to the issue of nontuberculous mycobacteria (NTM) colonization (*1*), which was not described in our study (*2*). Among 4,786 patients with NTM isolates treated at our hospital during 2000–2008, colonization was found in 76.9% (3,681), and only 23.1% (1,105) had NTM diseases according to the criteria of the American Thoracic Society and Infectious Diseases Society of America (*3*)*.*

Annual proportions of NTM isolates causing colonizations ranged from 29.2% in 2001 to 19.8% in 2007. During the study period, annual incidences of NTM colonization and disease increased from 6.6/100,000 inpatients and 2.7/100,000 outpatients in 2000 to 34.5/100,000 inpatients and 10.2/100,000 outpatients in 2008. *Mycobacterium avium* complex (MAC) was the most prevalent species, colonizing 1,282 (34.8%) of 3,681 patients. Annual proportions of MAC isolates causing colonization ranged from 20.0% in 2000 to 12.6% in 2006. Annual incidence of MAC colonization increased from 1.9/100,000 inpatients in 2000 to 12.3/100,000 inpatients in 2008; incidence of MAC disease also increased from 0.5/100,000 inpatients in 2000 to 2.1/100,000 inpatients in 2008. *M. abscessus*, the second most common species in our study (*2*)*,* caused colonization and disease in 669 and 155 patients, respectively. Annual incidence of *M. abscessus* colonization and infection also increased from 1.49/100,000 inpatients and 0.3/100,000 outpatients in 2000 to 7.0/100,000 inpatients and 1.9/100,000 outpatients in 2008.

Our study and a previous study in British Columbia (*4*) suggest that improvement in diagnostic methods would detect increased incidence of NTM, especially of MAC; most isolates identified in these studies were associated with colonization. We also demonstrated a gradual increase in the incidence of all NTM, MAC, and *M. abscessus* over time in Taiwan, which may be attributable to increasing vigilance and awareness of these bacteria as human pathogens and the increased population of immunocompromised patients. Thus, clinicians should consider diagnosing NTM diseases with sensitive and advanced laboratory methods because of the increasing population of patients at risk.

## References

[1] Hernández-Garduño E, Elwood RK. Increasing incidence of nontuberculous mycobacteria, Taiwan, 2000–2008. Emerg Infect Dis. 2010;16:1047.2050777810.3201/eid1606.100228PMC3086241

[2] Lai C-C, Tan C-K, Chou C-H, Hsu H-L, Liao C-H, Huang Y-T, Increasing incidence of nontuberculous mycobacteria, Taiwan, 2000–2008. Emerg Infect Dis. 2010;16:294–6.2011356310.3201/eid1602.090675PMC2958002

[3] Griffith DE, Aksamit T, Brow-Elliott BA, Catanzaro A, Daley C, Gordin F, An official ATS/IDSA statement: diagnosis, treatment, and prevention of nontuberculous mycobacterial diseases. Am J Respir Crit Care Med. 2007;175:367–416. 10.1164/rccm.200604-571ST17277290

[4] Hernández-Garduño E, Rodrigues M, Elwood RK. The incidence of pulmonary nontuberculous mycobacteria in British Columbia, Canada. Int J Tuberc Lung Dis. 2009;13:1086–93.19723396
